# Getting to the Root of the Problem: Supporting Clients With Lived-Experiences of Systemic Discrimination

**DOI:** 10.1177/24705470221139205

**Published:** 2022-11-21

**Authors:** Amy Bartlett, Sonya Faber, Monnica Williams, Kellen Saxberg

**Affiliations:** 1 Department of Classics & Religious Studies, 6363 University of Ottawa, Ottawa, ON, Canada; 2 School of Psychology, 6363 University of Ottawa, Ottawa, ON, Canada; 3 Department of Cellular and Molecular Medicine, 6363 University of Ottawa, Ottawa, ON, Canada

**Keywords:** marginalization, coping, advocacy, activism, social determinants of mental health, resilience, social capital, community, therapy

## Abstract

For many marginalized people, coping with discrimination is not a temporary condition. Rather it is endemic to living in a discriminatory society and a source of ongoing stress. In this paper, we explore the need to provide people struggling to cope with the skills to tackle not just the personal consequences of discrimination, but also to understand and address the root causes of their pain, and specifically the ones that lie outside of themselves. We propose using the concept of social capital to bring greater awareness among clients, clinicians, and society in general about the need to pair the treatment of personal distress with concurrent practices to understand and tackle larger systemic issues impacting their mental health. People with marginalized identities are often expected to find ways to cope with oppression and then sent back into a broken world, perhaps with stronger coping skills, but often ones which do not address the root cause or source of the pain, which is social injustice. We propose that it is therapeutically important to problematize, pathologize and address the systems and narratives that discriminate and cause people to need to cope, instead of focusing therapeutic interventions only on the internal resources of the person doing the coping.

## Introduction

The pain of coping is difficult for anyone to navigate. But unfortunately for people from marginalized communities, coping is often a way of life. Coping with marginalization and discrimination is stressful and shows up in many of the day-to-day experiences of marginalized folks, and in a myriad of ways. This paper was in part born out of an experience of one of the authors of this paper, who was in dialogue with a colleague wanting to launch a project to bring together ‘both sides’ of the queer ‘debate’ in the therapeutic community— essentially building a collection of advice for queer people that comes from people who are queer or queer allies, along with people who believe in conversion therapy and pathologize queerness.^1^ After hearing this and having concerns, she proposed that instead, it might be more appropriate to draft a two volume series: one volume in support of LGBTQ  +  (queer) people who are in the midst of managing their pains, written exclusively by queer people with lived experience, and another volume to support straight people who harbour stigmatizing and pathologizing views about ‘alternative’ sexual and gender identities, written by both queer and straight people to help build empathy and get at the actual root cause of queer pain, which is the fear and hate (whether intentionally or unintentionally wielded) that is harboured by homophobic practitioners and people in society. This suggestion was rejected by the editors.

The proposed reorientation was offered in an attempt to challenge the presumption that pathologizing a person's gender or sexual identity was a valid viewpoint to hold in a therapeutic context, and instead to encourage everyone to take responsibility for the pain they may cause themselves and others by denying the inherent value and legitimacy of people in society. By pathologizing homophobia instead of pathologizing queer identity or other marginalized identities, we can help create opportunities to treat not just the symptoms of discrimination and marginalization (i.e., the coping), but to also address the *external causes* of the pain marginalized people face.^1^

For unfortunately, marginalization and discrimination is not only something people cope with, but can be a source of stress and trauma. Holmes and colleagues^2^ describe a phenomenon called oppression-based traumatic stress which is the cumulative, traumatizing effects of marginalization from one or multiple sources, and they call upon the mental health community to formally recognize its sequelae. Mental health clinicians are trained to work with clients to help reorder the internal emotional wreckage of the aftermath of trauma and support a client's efforts to choose coping strategies that can move them towards a more internal healed state. However it is often unclear, even to psychologists, to what extent the *external* causes of discrimination can be addressed by any one individual, and how to confront these external causes. This incomplete understanding is due in part to the emphasis Western societies place on the individual rather than the community. However, among marginalized groups, the root cause of their pain is often coming from external rather than internal factors, and the ability to make transformative change for themselves and others can feel enormous and outside of their power or control.

To that end, and as a further anecdote, in the process of navigating the approvals for a recently published paper on coping,^3^ during the peer-review process another one of the authors was astonished to read some of the comments from reviewers about the prescriptions advanced in the manuscript for people facing discrimination. One particularly striking comment came in response to the following assertion in the paper that “How we define ‘functional coping’ depends on if the goal is to stop the distress caused by racist acts or to stop racism from occurring,”(^3^,p. forthcoming) to which the reviewer wrote, “It is not possible for Black individuals to stop racism from occurring so this definition makes no sense.” Stopping racism is in fact possible and imperative (e.g.,^4^), and this is best done by addressing both systemic and individual causes. On the systemic side, for example, this can involve exposing and dismantling unjust policies, and on the individual level it can involve teaching victims new ways to respond and engage, including strengthening the social fabric of community, and helping to build empathy in perpetrators when such actions feel safe and the person well-resourced to engage.^5^

With these two recent experiences, the authors of this paper felt compelled to explore how therapeutic practice can be better leveraged to empower people to engage in influencing the environment in which they operate, to engage community and connection as a coping strategy, and to explore the value of nurturing community resilience and social capital within marginalized communities as a therapeutically relevant technique for healing.^2^ While racist or homophobic actions perpetrated by individuals can be hugely damaging to targets, the reality is that much of the harm done to individuals by racism and discrimination comes from the communal or social level, where entire communities, and the systems and structures they have created to wield power, are used to dominate and discriminate against less powerful groups of people in their midst, both overtly and covertly.^7^ These acts and systems are associated with a host of stress-related physical and mental ailments in the oppressed, including depression, PTSD, generalized anxiety, hypertension, and substance use disorders.^8,9^

Whether it is residential schools designed to destroy Indigenous culture in Canada,^10^ implementing ‘don’t ask, don’t tell’ policies in the military,^11^ or the weaponized policy measures of redlining in the USA,^12^ it is possible to halt the injustice, repair the systems that created and enable the harm, and heal those who have suffered and been broken by these systems of oppression. When communities come together to heal wounds and right wrongs, this act of communion can be a powerful lifeline for individuals who have been hurt by those systems and are seeking better ways to cope with the pain they face.

In this paper we explore how the individual can synergistically work to heal themselves while supporting the transformation of external unjust systems to restore marginalized social capital and address the inequity in our societies. This is not meant to propose putting all of the work on those who are suffering, as we will see below, but instead to propose that there can be a virtuous cycle of individual and social therapeutic benefit to working for positive social change. A community's resilience is dependent on its social capital, which must be appreciated as a determinant of social mobility and social influence. Communities can become under-resourced, often depleted by structural racism and discrimination,^13^ and so too are the individual members of that community depleted and under-resourced as a result.

As individuals work to nurture community and social change as part of their healing practice, this supportive and generative act can provide emotional, psychological and structural resources in return, which individuals can rely on and build off of as part of their own healing journey. War tacticians have long understood that when one can deplete the social capital of a group with whom one is in conflict; they are that much easier to take advantage of.^14,15^ One can destroy a community by destroying the culture, language, nutrition, agency, cohesion, and educational opportunities of the current and next generations. This is what structural racism and systemic discrimination were designed to do. Taking action to counteract these forces is an act of agency and empowerment. Therefore we believe that individuals do indeed have the power to stop racism (e.g.,^4,16^), people who harbour discriminatory opinions can be held accountable for their discrimination, and marginalized people can exercise the power to create a better world by being supported in their efforts to take steps to impact the social structures that allow racism and discrimination to happen in the first place.

## Some Therapeutic Best Practices for Minority Coping

One of the most successful and healthy coping mechanisms to combat the stress of oppression is social safety and support (e.g.,^1,17^). Social support refers to the availability of one's network members who provide support, care, and love. Family, friends, intimate partners, coworkers, neighbors, and anyone who offers care and support for an individual are part of the social support network.^18^ Research has demonstrated that these human connections are protective against mental health concerns arising from discrimination.^19–22^ Further, social support may be particularly important for people who are part of marginalized groups. For example, due to a long and enduring history of institutional racism, and the resulting cultural mistrust,^23^ people of color may be less likely to utilize formal mental health care and may prefer relying on community support, which is also consistent with more collectivist values.^4,24,25^ When people share their life experiences of marginalization with those who have had similar experiences, they may feel a sense of connection and understanding (Williams et al., 2022),^17,26^ or even deeper kinship.^27,28^ Discussing experiences with others they can trust also provides an opportunity for emotional processing and examining maladaptive cognitions (i.e., internalized racism, sexism, homophobia, etc).

Self-care is critical for recovery from various physical and emotional traumas and experiences, and is required for sustained well-being.^29^ Self-care is a broad term, but here we specifically refer to self-focused behaviors for personal health and wellbeing. Further, some make a distinction between regular self-care and racial self-care, which is described as a tool for social justice and survival for marginalized communities, with a goal to promote self-determination, self-preservation, and self-restoration in an environment of ongoing oppression.^30^ Queer Black activist Audre Lorde notes, “Caring for myself is not self-indulgence, it is self-preservation, and that is an act of political warfare” (^31^, p. 125). Likewise, it could be argued that ‘loving oneself’ also means being intolerant or even angry at injustice, advocating for your rights and freedoms, and taking a stand against views and practices that pathologize and stigmatize one's authenticity and identity. Anger and advocacy has a role in ‘living well’ too.

Oppression and marginalization can also injure a person's sense of spirituality and connection with the divine. With respect to meaning making after trauma, Allen et al.^32^ note that spiritual resources and interventions that are in line with a person's beliefs can help people cope, heal, and grow. Religious rituals can be used to enact destroying the traumatized life and nurture an experience of a renewed and more meaningful life. Spirituality can be cultivated through seeking out and incorporating moments of awe into one's life, which can help restore physical and mental health. Looking at the stars, gazing at a natural wonder, or contemplating the vastness of time can trigger feelings of awe. Likewise, reflecting on the kindness and compassion of others can also inspire awe (an emotion termed ‘elevation’) and improve our mood.^33^

Techniques such as those described by Mosley and colleagues^34^ as well as other researchers described above, contribute to a person being able to successfully cope with minority stress by fostering resistance and resilience in the face of oppression (see Table 1). All of these coping strategies and techniques can be supportive to people coping with the pain and stress of marginalization and discrimination. What ties them together in part is the healing power of connecting to others to find support and resourcing– in short, accessing the social capital and strength of the community they are a part of. That said, while we can help and support each other to cope with living in this broken world, if we don't also try to fix what is broken — around us and within us — then we are missing out on the fundamental opportunity for genuine and sustainable healing. These strategies can help people find themselves, find each other and find community, and in so doing, people can be empowered to mutually cope, heal, connect, and take systemic-liberatory action.

**Table 1.** Techniques That Foster Resistance and Resilience in the Face of Oppression.

**Technique**	**Description**
Social support	The availability of others who provide support, care, and love such as family, friends, intimate partners, coworkers, neighbors, and anyone who offers care and support for an individual
Self-care	Self-focused behaviors for personal health and wellbeing
Racial self-care	A tool for social justice and survival for marginalized communities, with a goal to promote self-determination, self-preservation, and self-restoration in an environment of ongoing oppression
Spiritual resources and interventions	Resources and interventions that are in line with a person's beliefs such as religious rituals and other forms of spirituality
Storying survival	Sharing narratives, experiences, lessons learned
Art-ivism	Using creative art to advocate for liberation
Physical resistance	Putting one's body in risk in order to defend, free, or affirm marginalized people
Organizing	Designing objectives, defining results, and selecting tactics for anti-oppression related work
Teaching	Educating people and encouraging them to continue learning about anti-racism and de-colonizing approaches
Coalition-building	Creating, maximizing, and sustaining connections
Modeling/mentoring	Contact with another marginalized person on a one-on-one basis to confirm, support, and enhance collective health
Scholar activism	Participating in and contributing to activism informed by scholarship
Spacemaking	Creating physical or virtual venues for stigmatized groups to gather to heal, organize, and/or celebrate

## The Therapeutic Gap: Addressing the Root Causes of Systematic Discrimination for Minority Stress

While it is important to understand coping strategies to address the internalized pain of those who are suffering, when it comes to minority stress and discrimination, we argue that it is equally important to understand and address the external source of the pain. The roots of the pain a person is suffering from may have concurrent internal sources such as childhood trauma. However when it comes to minority stress, while experienced internally, the cause is external to the person, and therapeutic options for treating minority stress should include non-retraumatizing actions and efforts to hold the people, systems, and structures accountable for the pain that is being experienced.

When treating minority stress, we must start from the premise that being queer, or a woman, or racialized, or differently-abled is not wrong. We must understand that the pain the person is experiencing is not right or fair or okay, and feeling this pain is not inherent to their identity or worth as a human being. It is good and right to acknowledge this pain and admit to suffering.^35^ But we also do not need to accept it as an inevitable part of existing as a marginalized person in society.

The approach we propose goes beyond acknowledging the pain people are coping with, and seeks to understand the root cause of that pain and take therapeutic actions in the external world that are both internally and externally empowering and resourcing for clients. By addressing the external conditions that have created the pain they are experiencing– rather than accepting that this is just ‘how the world is’ and helping people try to make do with what they've got — this approach can help people access some of the best practices around coping with minority stress outlined above in Table 1, while also contributing to longer-term and sustainable change in their circumstances, making their external world a safer place to be in for themselves and others. Of course all of this must be approached with close attention paid to the resources and boundaries of the people who are already suffering in the miasma of homophobia (racism, misogyny, ableism, etc.), and should be engaged with in such a way so as to not retraumatize the person or put them in harm's way (see below). By helping clients safely engage in transforming not just their inner worlds but also external contexts, these efforts can ideally act as both a ‘cure’ for the pain they are currently facing as well as a preventative mental health intervention for future pains – both theirs and the pains of those in their community.

Ultimately, the work of healing minority stress does not only reside with the person experiencing pain. When it comes to racism and discrimination, everyone has a responsibility for healing themselves and their community, and must be actively engaged in doing so. For example, coming back to the queer book volume experience outlined in the introduction to this paper, it is not safe, just, or equitable of us to suggest that only queer folks bear the burden of advocating and coping with homophobia without also demanding that straight folks take a look at their own implicit and explicit biases, and how they are complicit in this pain. Demanding recognition of the pain caused is not meant as some form of schadenfreude or retribution, but instead as both an individual and societal balm. It is not possible to fix something that is invisible or unacknowledged. So similar to the problem of rape culture, for example, if we are only talking about what women should do to protect themselves from being raped without talking about the problem of men who rape and toxic masculinity, then we are ignoring the source of the problem and the chain of causation.

Critically confronting discrimination means engaging social justice strategies.^34^ Acting in community and making a meaningful contribution to anti-oppression and pro-justice causes around issues of structural injustice can be a healing act of agency and self-affirmation. Clinicians can help clients identify additional ways to respond to discrimination through social action. An important part of therapy for oppression-based stress and trauma should be helping the client develop self-efficacy in engaging in anti-oppression or social justice initiatives, and in ways that align with their values.

## The Role of Community in Coping

Empowering individuals to engage in their communities can be a form of healing. This investment contributes to a virtuous cycle, because strong communities also help heal individuals. Community resilience is essential for helping individuals within a community to cope with crises, and can be defined as the collective ability of a community to cooperatively cope with external stressors and resume efficient normal daily life in the aftermath of a crisis.^36^ The more *social capital* a community possesses, the more resilience it will have to weather disasters, threats and upheaval.

According to papers by Aldrich and colleagues, social capital plays a role in generating benefits beyond individuals at the neighborhood and community level.^29,30^ For communities in crisis, social capital networks provide a lifeline to essential resources, which include but are not limited to information, financial resources, nourishment, child care and emotional and psychological support.^29^ However, social capital publications in regards to coping, particularly in regards to marginalized and racialized communities, are sparse. There are fewer metrics to help us understand the role of social capital than other economic or demographic factors in measuring community and individual resilience. However there are surveys that measure the effect of social capital in communities.^29^ Specifically, there are three types of social capital described within the literature. Within each variant, different strengths of relationships and network compositions are recognized. Therefore the amount of each type of social capital in any particular community results in different experiences and outcomes for that community and the individuals within it.^29^

These three recognized types of social capital include bonding, bridging, and linking (see Table 2). Marginalized communities often have distinct types and levels of these three types of social capital compared to advantaged and White communities. And, as social capital can be both increased or undermined, even before these concepts were described or there were scales to measure them, those belonging to powerful social groups have sought to destroy the social capital of subordinate communities. A wide variety of studies have demonstrated that social capital can be increased through community currency, social events or community architectural structures,^29,31–34^ while conversely, social capital can be damaged by things such as destroying community space.^35^

**Table 2.** Types of Social Capital (from^29^).

**Type**	**Groups**	**Characteristics**	**Example**
Bonding social capital	Experienced between friends or family, and among individuals who are emotionally close, resulting in tight bonds to a specific group	Characterized by high levels of similarity in attitudes, resources, demographic characteristics, shared information sources	An individual experiences an act of racism or homophobia and is emotionally distressed. They can reach out to friends and family for support, and their friends and family are likely to empathize and understand their experience, as well as care more deeply about the pain they are experiencing
Bridging social capital	Experienced between individuals or acquaintances loosely connected across social groups, such as class or race	Characterized by higher demographic diversity. Provides individuals with novel information and resources that promote societal advancement – i.e. job opportunities; utilized in organizations including political, civic and academic institutions, educational and religious groups, or sports and interest clubs	An individual is trying to find employment in their field of expertise. With a high level of bridging social capital, instead of simply applying to job advertisements and hoping for the best, a person with high bridging social capital can tap into their professional network and connect with someone they know in the organization to let them know they are applying, get advice on their application, and perhaps even an in-house reference. All of these would be supportive of getting the application noticed, and potentially securing the position
Linking social capital	Experienced between regular citizens and those in power	Characterized and embodied in norms of respect and relationships of trust between people who are interacting across institutionalized power or authority gradients in society	An individual expresses their concerns to a government representative about a new proposed construction project that inadvertently limits their access to public transport. The government representative relays those concerns to other government officials and to the project managers, who modify the final plans to address the oversight and avoid harming the community.

Some marginalized communities use community coping mechanisms which are unique and adapted to the specific stresses faced by their societies. Social justice movements such as the Montgomery Bus boycott,^36^ the March on Washington (MLK),^37^ the Million Man March,^38^ the Dakota Access Pipeline protests,^39^ the Stonewall riots,^40^ and most recently Black Lives Matter^41^ protests, all represent communal activities undertaken by Black, Indigenous and Queer Americans that pull together the three forms of social capital (bridging, bonding and linking) but are rarely recognized as a form of coping.^12^ However, this kind of protest creates agency, and manifests in a way that both grows social capital and can be highly therapeutic. The form of coping that encompasses public protests, social justice movements and marches should be acknowledged as a legitimate therapeutic tool rather than being seen simply as a dissent against governmental authority.^12^ Ultimately, mental and emotional health for most people cannot be achieved in isolation. And the community being sought does not have to be in a homogenous enclave. Even a bowling team or a knitting club can serve as community. Somewhere out there, there are people who think like the client, who have shared experiences, who want similar things for themselves and the world around them, who want to connect, to create and to grow. Part of the role of the therapist can be to help the client understand what kind of social capital– and what kind of community– would be most beneficial to their healing process.

In short, social capital is an underutilized therapeutic resource that strongly influences resilience at both the communal and individual level. Coping paradigms should look beyond individual actions as one of the most effective vectors of coping, and instead look to those being used more frequently in racialized communities.^3^ Just like other forms of capital, social capital can be nurtured or destroyed. Placing more focus on enhancing social cohesion and deepening trust within and among communities will lighten the burden on individuals by providing them with a well of good-will which can be drawn on in a personal or social crisis, as well as providing ongoing stability and connection to improve individual health and resilience.

### Social Capital in Racialized and Marginalized Communities

The challenge in cultivating social capital and community resilience within marginalized communities is that, unfortunately, marginalized communities often exist in a state of constant crisis. The stash of social capital is not being used to periodically weather a rare catastrophic storm. Rather, hurricane level winds are constant, and the meager store of ‘provisions’ are being constantly scattered to the winds or plundered by wild animals. The history of racialization in the US or the treatment of Indigeous Canadians, for example, shows how carefully and deliberately entire communities have been strangled in an attempt to completely deprive them of social capital, since a reduction in social capital (as with all kinds of capital) represents a way to stunt community power (i.e., Greenwood in Tulsa; the *Indian Act* in Canada^42,43^). In these and many other examples, the source of the depletion of social capital is not a natural disaster or momentary crisis, but rather due to unfettered and ongoing structural racism and discrimination.

Systemic discrimination results in injustice in part by dictating which resources and opportunities are permitted to be accessed by whom. Therefore, structural bias serves as a gatekeeper for both community-level and individual access to all types of capital (e.g., cultural, financial, and social).^12^ The concept of social capital allows us to typify and quantify the value of networks and the norms within these networks which give rise to cooperation and reciprocity. Social capital allows us to advance not only collective and individual goals but also to leverage the power of community (macro) super-structures.^12^ Understanding that marginalized and racialized communities often face a deficit in linking their social capital to the systems that serve the community, focusing on measures targeted at building and maintaining these connections will increase the power of these communities to make sustainable positive changes to their contexts (ie. reducing environmental racism, increasing local infrastructure such as museums, parks and community centers, etc…).

The lack of discussion of social-capital-deficit as a driver of marginalization, discrimination and racial disparity in North American communities, and in therapeutic contexts, is telling considering the role of social capital as a mechanism to redistribute power from over-arching community structures such as the police force, the hospitality industry, housing labor markets, healthcare, and into local communities.^12^ Social capital as a concept to help explain social disparities has not been sufficiently explored by researchers and clinicians as an effective lens with which to make visible the often invisible social strands that tie communities and individuals together, or exclude them.

## Building Community and Social Capital as a Coping Strategy: Recommendations for Clinicians and Therapists

In summary, the pain that marginalized people face is experienced internally (and can be deeply damaging), but the source of this pain does not reside in their sexual orientation, race, gender, ability or identity — it comes from the external environment and the rampant social stigma, hate, othering, and pathologizing of their experience (and sometimes existence). Social capital can serve as a helpful lens with which to understand the connection between an individual, their marginalization and the role of community as they heal. And actively engaging with one's community and society to create equitable change can play an important role in addressing individual stress while also contributing to a longer-term transformation of the root causes of the pain they are facing.^44,45^ This engagement can serve those who are suffering in the moment. But in the end, we all play a role in changing the external environment as the people suffering work to heal their internal experience of the broken world in which they live (^46^; Williams et al., 2022). So with all of that said, how can therapists use activism, community and social capital as therapeutic tools to support marginalized clients who are struggling to cope?

First, it is essential for individuals in marginalized communities– and the therapists who serve them– to understand how systems of oppression directly limit their social capital in order to take corrective action and properly resource themselves. Educating oneself and marginalized patients on social capital and community can provide a sense of validation and agency, as well as equipping clients to engage in activism efforts in communion with others. As long as individuals remain ignorant to the ways that oppression operates, their and others’ actions will continue to contribute to causing pain, whether intentionally or unintentionally. Furthermore, the process of educating oneself and others can itself act as a coping mechanism. Understanding systemic discrimination can be validating of the pain being experienced. Educating others can also serve as a form of active involvement in fighting against oppression at both an individual and community level. This act of resistance through education can instill a sense of purpose, cementing it as a valuable coping skill, just as protest encourages coping through a similar mechanism.

Second, efforts must be taken to ensure that the activism efforts that are pursued are done so in community with others. Active confrontation with hostile systems must be done with community support and not simply individual involvement, for a few reasons. For one, engagement with the systems of oppression that are causing pain can be retraumatizing. Approaching this engagement with the support of others can provide avenues to process, understand and resist potentially traumatizing encounters with others. Working with others who are already engaged in activism can help develop skills and learn about how to engage well in creating positive change in the community. Additionally, not only is this communal approach consonant with existing best practices when it comes to coping (see Table 1 above), but it can be also more effective to the overall goal of social equity: while both can be therapeutically relevant, when it comes to social change, strategies that address structural problems are often more beneficial in the long term than many small, person-to-person battles.

Through activism it is possible to address the larger sources of oppression, and this can provide a satisfying and therapeutic coping experience which can reap long term benefits not just for the individual involved but for the whole community (e.g., ^15^). Contributing to social justice initiatives that dismantle oppression can also serve as a coping tactic of agency and self-affirmation (; Williams et al., 2022, ).^47,48^ Certain forms of activism furthermore seem to have specific mental health benefits.^56–58^ Ensuring that the activism-oriented coping option chosen allows the client to reclaim their identity and dignity is essential. Also consider that activism comes in many forms and may or may not involve formal protests.^59^ Clients can look for opportunities to promote prosocial change in their personal environments (work, school, community) through any number of means.^48^

Finally, clinicians have a responsibility to be allies as their clients navigate minority coping stress instead of watching the sidelines as their patients cope (Williams et al, 2022). Ratts et al.^50^ outlines several ways that clinicians can use a Multicultural and Social Justice Counseling Competencies (MSJCC) framework to help build a more equity-focused engagement with marginalized clients. Some suggested tactics include:

- *Therapist self-awareness*: Seeking out learning opportunities and intentionally building an awareness of your own experience of your social identities, the status of your social group vis a vis other groups, understanding power and privilege, as well as exploring your own assumptions, biases, values and beliefs about your client's marginalized identity
- *Understanding a client's world view and how it intersects with your own*: Clinicians should also cultivate an understanding of their client's assumptions and attitudes about society, and explore their lived experience of power, privilege and oppression in their lives. Validating their lived experience is key to building an understanding of how your client experiences the oppressive external environment in which they operate. It is important to take the final step of analyzing how your worldview and that of your client influence and interact as part of the therapeutic relationship.
- *Engaging in justice-oriented counseling and advocacy interventions*: Examples abound and may include using less-hierarchical therapeutic interventions (ie: Functional Analytic Psychotherapy) with marginalized clients; actively collaborating with social justice organizations to contribute to the work towards dismantling oppression; or offering pro-bono or sliding scale rates for people experiencing inequity in your community

Whatever interventions a therapist engages, we recommend that as someone working with marginalized clients, it is essential to be actively engaged in understanding the system and structures impacting your client's mental health, and consider social capital and its influence in order to provide effective support to your client. More research is needed to better understand how to navigate the potential benefits and risks of engaging in community activism as a therapeutic tool. This also extends to better understanding how this tool might be leveraged across different age ranges, different geographical contexts (urban versus rural), etc… However, fundamentally, a therapist who is going to discuss the possibility of direct social action also needs to inform the client that this will take courage, and that while courage can bring moral and emotional growth, the very definition of the word also means that there will be risk. Risk always brings the potential for injury. However there is no way to gain resiliency, overcome fear, or cultivate bravery without real risk. Facing fears of all kinds requires risks, and the potential risks and benefits of social justice activism must be clearly and comprehensively discussed and disclosed.

## Conclusion

Coping with marginalization does not have to be faced alone– and it is often most effectively tackled when it is faced with others. In community there is strength, but that community must be witnessed, cultivated, protected, and supported collaboratively by the people (and their allies) who are seeking equity for the lived experience of marginalization. Advocacy and community engagement can act as a healing modality for treating minority stress, racial stress and the trauma of discrimination and oppression. However, more research into social capital and the role of community in coping and oppression-based stress and trauma is needed. The work of our profession is not just to improve our understanding of the coping mechanisms of those who are marginalized– without addressing the root cause of their suffering, we perpetuate the inequity and marginalization they face, and continue to lay all of the responsibility for healing on the doorstep of those who are already under-resourced and suffering. There is a great deal of work to be done to improve the lived experience of marginalized people in our society, and the work of healing these wounds should not be left solely to the people suffering them, but must also be born by those who are participating in and perpetuating the systems and structures that lead to marginalization in the first place. Acknowledging this shared responsibility is reconciliation, social justice and allyship at its core.^62,63^

## Notes

1. We use the reclaimed term “queer” to inclusively refer to lesbian, gay, bisexual, transgender, intersex, asexual, two-spirit, and more (LGBTQIA2S+) individuals.

2. The critical academic term that describes quantifiable aspects of community power is often referred to as social capital. For the purposes of this paper, we define social capital as, “networks together with shared norms, values and understandings that facilitate cooperation within or among groups” (6, p 41).
